# Assessing the COX-2/PGE2 Ratio and Anti-Nucleosome Autoantibodies as Biomarkers of Autism Spectrum Disorders: Using Combined ROC Curves to Improve Diagnostic Values

**DOI:** 10.3390/cimb46080513

**Published:** 2024-08-08

**Authors:** Afaf El-Ansary, Hanan A. Alfawaz, Abir Ben Bacha, Laila AL-Ayadhi

**Affiliations:** 1 Autism Center, Lotus Holistic Alternative Medical Center, P.O. Box 110281, Abu Dhabi 23251, United Arab Emirates; 2 Department of Food Science and Nutrition, College of Food & Agriculture Sciences, King Saud University, P.O. Box 22452, Riyadh 11495, Saudi Arabia; halfawaz@ksu.edu.sa; 3 Department of Biochemistry, College of Science, King Saud University, P.O. Box 22452, Riyadh 11495, Saudi Arabia; aalghanouchi@ksu.edu.sa; 4 Department of Physiology, Faculty of Medicine, King Saud University, P.O. Box 2925, Riyadh 11461, Saudi Arabia; lyayadhi@ksu.edu.sa

**Keywords:** autism, auto-immunity, anti-nucleosome autoantibodies, cyclooxygenase-2, prostaglandin, Wnt signaling, glutamate excitotoxicity

## Abstract

Autism spectrum disorder (ASD) is a neurodevelopmental condition marked by restricted and repetitive behaviors as well as difficulties with social interaction. Numerous studies have revealed aberrant lipid mediators and autoimmunity as a recognized etiological cause of ASD that is amenable to therapeutic intervention. In this study, the relationship between the relative cyclooxygenase-2/prostaglandin E2 ratio (COX-2/PGE2) as a lipid mediator marker and anti-nucleosome autoantibodies as an autoimmunity marker of ASD was investigated using multiple regression and combined receiver operating characteristic (ROC) curve analyses. The study also sought to identify the linear combination of these variables that optimizes the partial area under the ROC curves. There were forty ASD children and forty-two age- and gender-matched controls included in the current study. Using combined ROC curve analysis, a notable increase in the area under the curve was seen in the patient group, using the control group as a reference group. Additionally, it was reported that the combined markers had improved specificity and sensitivity. This study demonstrates how the predictive value of particular biomarkers associated with lipid metabolism and autoimmunity in children with ASD can be measured using a ROC curve analysis. This technique should help us better understand the etiological mechanism of ASD and how it may adversely affect cellular homeostasis, which is essential to maintaining healthy metabolic pathways. Early diagnosis and intervention may be facilitated by this knowledge.

## 1. Introduction

Autism spectrum disorder (ASD) is a neurodevelopmental disorder that affects the pediatric population. Worldwide, the prevalence of ASD is estimated to be 1 in 100 children. Over time, prevalence estimates have risen and show significant variation both within and between sociodemographic categories [1].

Classical approaches to ASD usually concentrate only on neuro-functional aspects. According to scientific studies, ASDs affect a variety of physiological systems, including the immune system, the sensory-motor system, and the gut–brain axis [2]. Recent research suggests that connective tissue, which connects all bodily organs and systems, may have a role in the development of ASD and its associated co-morbidities. As a result, in autism, inadequate connection might cause increased sensitivity to environmental signals. The following interpretive model, known as the “connectivome theory”, considers variations in connective elements of shared mesodermal origin observed in various organs, as well as the evaluation and interpretation of ASDs by emphasizing somatic aspects and co-morbidities [3]. Research has established a comorbidity and co-occurrence between genetic connective tissue disorders—like Ehlers–Danlos syndromes and hypermobility spectrum disorder—and autism within the same families [4,5].

Growing data suggest that immunological dysfunction may contribute to the etiology of ASD. Maternal autoantibodies and inflammation during pregnancy significantly increase the risk of having an autistic child [3]. Individuals with ASD have much more immune-mediated co-morbidities than the general population, including gastrointestinal problems and dysbiosis. Higher levels of autoantibodies and neuroinflammation have been detected both in vivo and in postmortem brain tissue. Although the quantity of evidence linking autoimmunity, immunological dysfunction, and ASD is fascinating, it raises fundamental questions about the role of immune system failure in the development of ASD [4]. Many other factors, such as genetic predisposition, environmental conditions, and timing of exposure during development, are believed to have a role in the many symptoms and manifestations of autism [6].

Autoantibodies can pass the blood–brain barrier and produce immune complexes that damage neurological tissue. Some maternal autoantibodies could be regarded as biological indicators for autism, and they may play a role in the pathology of this disorder [7,8].

Recent evidence suggests that the nucleosome, a fundamental unit of chromatin and a natural byproduct of cell death, plays an important role in various autoimmune disorders because it is a primary target autoantigen for autoantibody-mediated tissue lesions [9,10].

Autoantibodies cause inflammation and injury to several organs, either directly or through the creation of immune complexes [11]. The presence of anti-nuclear antibodies is the primary diagnostic test for systemic lupus erythematosus (SLE) and many other connective tissue diseases [12]. The contributions of the primary participants in the pathogenic autoimmune response, namely, T cells, B cells, dendritic cells (DCs), and macrophages that are abnormally hyperactive in lupus, are dependent on elevated cyclooxygenase-2 (COX-2) expression and activity, which is similar to inflammatory cells in target organs [11].

Up-regulation of COX-2, and hence prostaglandin E2, is required for DCs’ survival, maturation, and activation [13,14], and DC’s hyperactivity in lupus results in immunogenic presentation of autoantigens [15]. Not only T cells but also DCs and macrophages in an SLE rodent model constitutively hyper-expressed COX-2 and NF-κB activation, which is necessary for the functioning of these cells of the lupus immune system [14].

There is debate over the contribution of neuroinflammation and the immune system to the development of autism. Immune theories were not well supported until recently, but studying the relationship between immunological response and neuroinflammation may have significant clinical and therapeutic consequences. A ratio is typically defined as one variable divided by another. Ratios are commonly employed in biological sciences and are suggested for their higher predictive values than independent variables [16]. In relation to this, it was interesting to find the relationship between the COX-2/prostaglandin E2 (PGE2) ratio as a marker of inflammation and anti-nuclear antibodies as a marker of abnormal immune response in the pathophysiology of ASD and its co-morbidities [16,17].

Abruzzo et al. emphasized the importance of employing receiver operating characteristic (ROC) curves as a good statistical tool for discovering biomarkers that are sufficiently sensitive and specific for early ASD diagnosis [18]. Although more research is needed to determine their utility in predicting, evaluating, and assessing treatment approaches, ROC curves highlight the most statistically significant differences between patients and controls.

The goal of this work is to better understand the pathophysiology of ASD by analyzed the diagnostic utility of anti-nuclear autoantibodies and the COX-2/PGE2 ratio in the blood plasma of patients with the disorder. This will be achieved by using both independent and combined ROC curves.

## 2. Materials and Methods

The ethical committee of medica collage, King Saud University, accepted the study protocol in accordance with the most recent Declaration of Helsinki. The study recruited 40 autistic patients and 42 age- and gender-matched controls. All subjects provided written informed consent through their parents and agreed to participate in the study. The study participants were enrolled at the ART Center (Autism Research & Treatment Center) clinic. The ART Center clinic sample population included children diagnosed with ASD. All study participants’ ASD diagnoses were confirmed using the Autism Diagnostic Interview-Revised (ADI-R), Autism Diagnostic Observation Schedule (ADOS), and 3DI (Developmental, Dimensional Diagnostic Interview) procedures. The study included autistic children aged 2–12 years old. All were negative for the fragile X gene study. The control group was recruited from the pediatric clinic at King Saud medical city, and their mean age ranged from 2 to 14 years. Subjects were excluded from the study if they showed dysmorphic characteristics or were diagnosed with fragile X or other serious neurological (e.g., seizures), mental (e.g., bipolar disorder), or other medical problems.

All individuals were assessed for present and previous physical illnesses through a parental interview. Children with known endocrine, cardiovascular, lung, liver, kidney, or other medical conditions were excluded from the study. All of the patients and controls in the study ate a similar but not identical diet, and none of them followed a special high-fat or fat-restricted diet.

### 2.1. Blood Sampling

Following an overnight fast, a competent technician drew blood samples from participants into 3 mL blood collection tubes containing EDTA. Blood was collected immediately and centrifuged at 3000× *g* for 20 min at 4 °C. The plasma was separated, divided into three 0.5 mL aliquots to avoid multiple freeze–thaw cycles, and stored at −80 °C until use.

### 2.2. Biochemical Assays

#### 2.2.1. Anti-Nucleosome-Specific Antibodies

The ELISA technique (EUROIMMUN Medizinische Labordiagnostika AG, Lübeck, Germany) was used for this study; samples were run in parallel on the same run with the same internal standards, randomly chosen, and blinded, with the expectation that any antibodies to highly purified human nucleosomes (anti-nucleosome-specific antibodies) present in the diluted serum would bind in the microwells. Unbound serum antibodies were removed by washing the microwells. A conjugate/antibody/antigen complex was formed when the patient’s antibodies were immunologically linked to the horseradish peroxidase-conjugated anti-human IgG. The substrate was hydrolyzed to produce a blue hue when it was washed in the presence of bound conjugate. When an acid was added, the process was stopped and a yellow byproduct was produced. Photometrically, the intensity of this yellow color was measured at 450 nm. The quantity of color corresponded exactly to the number of IgG antibodies in the original sample. All samples were examined twice in two separate trials to maximize accuracy, evaluate inter-assay differences, and guarantee repeatability of the obtained outcomes (*p* > 0.05). There was no discernible interference or cross-reactivity.

#### 2.2.2. Cyclooxygenase-2 (COX-2)

A quantitative sandwich enzyme-linked immunosorbent assay (ELISA) kit from CUSABIO (8400 Baltimore Avenue, Room 332, College Park, MD, USA) was used to measure the levels of COX-2. With a minimum detectable dosage of 0.31 ng/mL, the measurement was carried out in accordance with the instructions supplied by the manufacturer.

#### 2.2.3. Prostaglandin E2

A study kit from USCN Life Science (Wuhan, China) was used to quantify PGE2. This assay uses the competitive inhibition enzyme immunoassay method, in which a monoclonal antibody specific for human PGE2 was pre-coated on a microplate. Usually, PGE2 can be detected at a minimum of less than 1.78 pg/mL.

#### 2.2.4. Statistical Analyses

The study evaluated data with IBM SPSS software version 22.0 (IBM Inc., Armonk, NY, USA). The Shapiro–Wilk Test was used to establish data normality in each group. The results were presented as the minimum, maximum, and median. The Mann–Whitney Test was used to compare two nonparametric groups, and *p*-values ≤ 0.05 indicated a significant difference. The Spearman rank correlation coefficient (R) was utilized to link various nonparametric variables. Logistic Regression analysis for the patient group using one variable as the dependent variable and the second as the independent variable was performed using the Enter method.

The odd ratios (ORs) from the logistic regression analysis show the correlation between the biomarkers and clinical state in the combined receiver operatic characteristic (ROC) curves. ROC curves were created for all logistic regression models. A nonparametric method was used to compute the area under the curve (AUC) for each marker combination. In logistic regression, odds ratios larger than one signify “positive effects” since they raise the likelihood. Due to their tendency to lower the probabilities, those between 0 and 1 are referred to as “negative effects”. A ratio of odds of exactly one indicates “no association”. A ratio of odds cannot be smaller than zero. The area under the curve (AUC) is a useful statistic for determining the predictive power of biomarkers. An effective way to evaluate the predictive power of biomarkers is to look at their AUC. A curve close to the diagonal (AUC = 0.5) has no diagnostic significance, whereas an AUC value close to 1.00 indicates a very good predictive marker. There is always a biomarker with appropriate specificity and sensitivity values when the AUC value is near 1.00 [19]. When looking at potential biomarkers for ASD, high sensitivity suggests that most patients will have ASD diagnosed; high specificity, on the other hand, means that healthy people will hardly ever test positive for the variable being studied. Additionally, adding two different markers in an ROC curve analysis typically boosts their specificity [19], indicating that using a panel of variables rather than a single variable may be very beneficial as a diagnostic tool.

## 3. Results

Demographic data are presented in Table 1. Forty children with ASD and forty-two age-matched control children were included in this study. All participants were males.

Table 2 describes the comparison between the control group and patient group for each parameter using the Mann–Whitney Test (nonparametric data). A significant decrease in COX/PGE2 ratio (−60.01% lower) together with a significant increase in anti-nucleosome autoantibodies (268.69% higher) values, with *p* < 0.001 for both variables, can be easily noticed.

Table 2 describes a comparison between the control group and the patient group for each parameter using the Mann–Whitney Test (nonparametric data).

**Figure 1:**
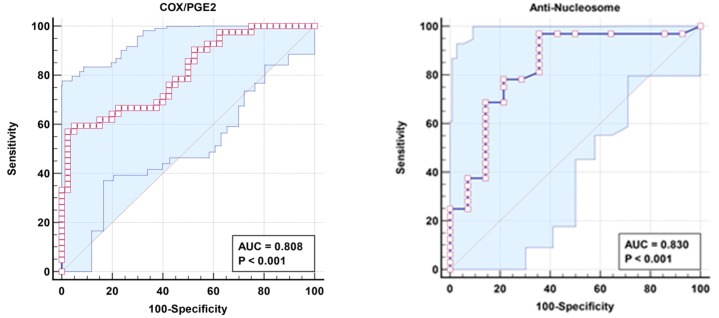
ROC curve for independent COX/PGE2 and anti-nucleosome of patient group according to control group.

**Figure 2:**
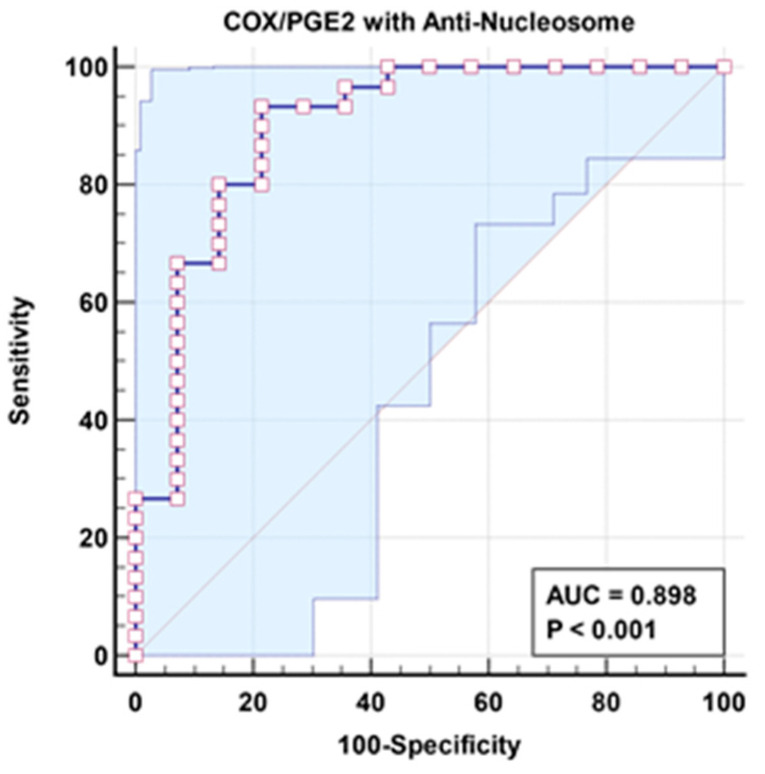
Combined ROC for COX/PGE2 with anti-nucleosome of patient group according to control group.

Table 3 demonstrates a non-significant negative correlation between the COX/PGE2 ratio and anti-nucleosome autoantibodies as markers of aberrant lipid metabolism and autoimmunity, respectively. The increase in the independent AUC for COX/PGE2 and anti-nucleosome autoantibodies from 0.808 and 0.83, respectively (Table 4 and Figure 1), to 0.898 when combined (Table 5 and Figure 2) could easily demonstrate the integrative contribution of both signaling pathways as etiological mechanisms of ASD.

Table 4 describes the Logistic Regression Test for the patient group as a dependent variable, with (COX/PGE2 and anti-nucleosome) as independent variables using the Enter method.

Table 6 presents the results of fitting the multivariate model, including a test of significance for each predictor while controlling for the other variable. This proves that the effect of COX-2/PGE2 and nucleosome autoantibodies as predictors of autism using standardized odds ratios (ORs) in the multivariate model differs in order and magnitude from that in the univariate model.

## 4. Discussion

Recent research reveals that ASDs are caused by combinatorial molecular alterations that affect metabolism, synapse and circuit activities [20].

The findings of this study highlight the importance of logistic regression as a simple clinical tool that holds great promise for aiding in the identification of ASD. According to the ROC analysis, the combination of anti-nuclear autoantibodies and the COX-2/PGE2 ratio resulted in significantly higher AUCs, the highest sensitivity, and the highest specificity for the diagnosis of ASD. This may make it easier to understand and interpret how autoimmunity and impaired lipid metabolism contribute to the pathophysiology of autism.

Prostaglandins E2 (PGE2) is an endogenous lipid molecule that plays an important role in normal brain development. COX2 is the primary regulator of PGE2 production. Normal COX2/PGE2-mediated signaling is involved in fundamental brain functions such as dendritic spine formation, synaptic plasticity, and memory and learning [21,22,23]. Emerging clinical and molecular research provides persuasive evidence that aberrant COX2/PGE2 signaling is linked to ASD [24]. It is well accepted that inflammation stimulates COX-2-catalyzed PGE2 production in CNS neuronal cells and is related to inflammatory pain hypersensitivity, a well-known feature of ASD [25,26,27]. Moreover, a link between aberrant COX2/PGE2 signaling and autism-related behavior in (COX)-2 mice, adding to existing clinical and molecular data, points to this pathway as a potential candidate for autism [24,28].

Considerable progress has been made in understanding the function of enzymes in health and disease, based on Michaelis Menten Km, the concentration of a substrate needed to reach half of the maximal rate, and other kinetic characteristics [27]. Changes in these parameters in wildtype versus mutant models, or active versus inhibited models, and variations in substrate or product concentration allow for several important comparisons between enzymatic activity in ill and healthy individuals.

The increase in independent COX2 and PGE2 [29] together with the significant decrease in their ratio (COX2/PGE2) as marker of aberrant lipid metabolism signaling in autistic individuals compared to the controls (Table 2) could be explained on the basis of altered COX-2 enzyme kinetics in ASD patients. It is interesting to mention that, as the Km (Michaelis constant) of an enzyme, relative to the concentration of its substrate under normal conditions, permits the prediction of whether or not the rate of formation of a product will be affected by the availability of the substrate, the significantly lower COX-2/PGE2 ratio in ASD patients recorded in the current study could help to suggest a COX-2 enzyme with a remarkably lower Km and higher affinity to AA (Arachidonic acid) as a substrate. This suggestion could find support by considering various abnormalities in key components of the COX2/PGE2 pathway due to both genetic and environmental influences which have been implicated in clinical studies on ASD [30,31]. For instance, increased ratios of AA to omega-3, decreased total AA, and increased PGE2 levels have previously been reported in blood samples of human patients with ASD [32,33,34].

The mechanisms underlying the increase in anti-nucleosome-specific autoantibodies in ASD patients (Table 2) remain unknown. Given the prevalence of nucleosomes in several autoimmune disorders, such as SLE [35], it has been postulated that substantially accelerated rates of apoptosis [36], as well as faulty processing of apoptotic cells, may contribute to autoantibody formation [37]. Nucleosomes may also stimulate lymphoproliferation and IgG synthesis in splenic B cells, as well as the generation of interleukin 6, a confirmed biomarker of ASD [38]. Altering these specified parameters confirms the role of apoptosis and neuroinflammation pathways in autism etiology [38].

In an attempt to explain this combined signaling (Table 5 and Figure 2) and how it could be related to ASD clinical presentation, it is interesting to note that dysfunction of β-catenin (β-cat) as a component of Wnt signaling leads to impaired social interaction and increased repetitive behaviors, as well as altered expression levels of genes linked to ASD in humans [20]. Previous research indicates that PGE2-dependent signaling can join with the canonical Wnt signaling pathway at the level of β-catenin via EP1-4 receptors (prostaglandin E2 receptor 1) [39]. PGE2 regulates the effects of Wnt signaling through cAMP/PKA activity, and it may directly regulate β-catenin destruction by the nucleosome and subsequent proteins available for transcriptional activation, which promotes gene expression and cell proliferation.

Based on the fact that excessive β-cat enhances dendritic branching, spine density, and synaptic function in cultured hippocampal neurons, indicating potential excitability imbalance, the decrease in COX-2/PGE2 and the increase in anti-nucleosome autoantibodies reported in the present study could interrupt β-cat’s destructive mechanism, leading to accumulation of β-cat as contributor to glutamate excitotoxicity as an established etiological mechanism of ASD [39,40,41]. Furthermore, it might be connected to abnormalities in connective tissues in ASD, which manifest as significantly elevated anti-nucleosome antibodies. Intestinal dysfunctions, malabsorption, and leaky gut syndrome are all conditions that may be connected to decreased intestinal connection [3]. This explanation could find support from a previous study by Kharrazian et al., which reported that increased anti-nucleosome autoantibodies are related to leaky gut as a co-morbidity in individuals with ASD and other autoimmune disorders [42,43].

Odds ratios larger than one in logistic regression represent “positive effects” because they enhance the likelihood. Those between 0 and 1 are referred to as “negative effects” because they tend to lower probabilities. A ratio of odds equal to one means “no association”. The ratio of odds must be smaller than zero. Based on this, both the negative correlations presented in Table 3 and the ORs presented in Table 5 could help to confirm the contribution of increased anti-nucleosome autoantibodies and decreased COX-2/PGE2 in the etiology of ASD in these positive and negative associations, respectively.

Based on this, the significant alteration of COX-2/PGE2 and anti-nucleosome autoantibodies (Table 2) could be related to glutamate excitotoxicity as one of the major etiological mechanisms of ASD. Carlson et al. [44] hypothesized that the inducible isoform of the COX enzyme, known as COX-2, may mediate a significant relationship between neuroinflammation and glutamate-mediated excitotoxicity in neurological illness. According to their model, oligodendrocytes that produce COX-2 may be more vulnerable to glutamate-mediated excitotoxicity.

This can find support through considering the fact that offspring born to female SLE patients had a higher frequency of cognitive problems than the controls [45,46,47]. While antibodies cannot pass through the mature blood–brain barrier into the adult brain, they can pass through the immature blood–brain barrier in developing embryos [44]. Research has demonstrated that certain kinds of anti-DNA antibodies can bind to fetal neural receptors, leading to excitotoxicity, which kills neurons [48,49,50].

**Figure 3:**
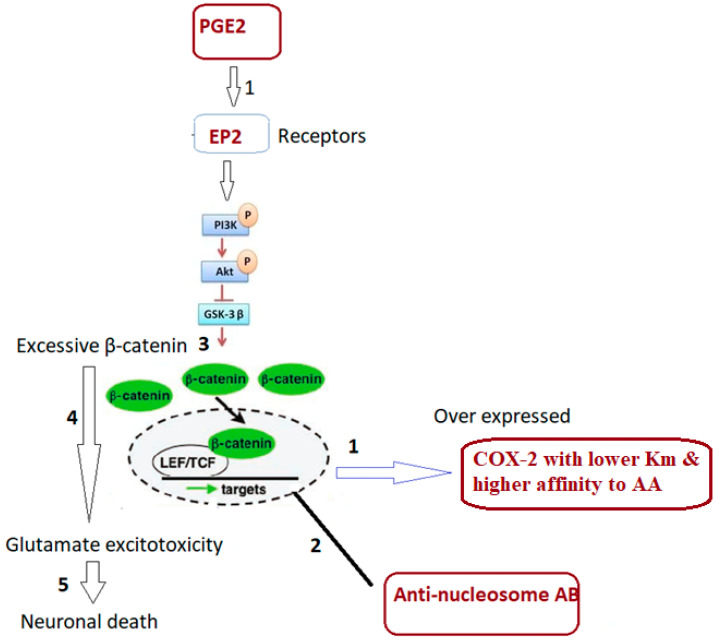
Illustrated mechanism of the integrative role of a lower COX-2/PGE2 ratio as a marker of impaired lipid metabolism (1) and higher anti-nucleosome autoantibodies as a marker of autoimmunity (2) in relation to abnormal Wnt signaling (3,4) and glutamate excitotoxicity (5), collectively leading to neuronal death in individuals with ASD. The relative contributions of these signaling pathways (1–5) may account for the heterogeneity and the diverse symptomatology seen in individuals with autism.

Within the context of canonical Wnt signaling, β-catenin is phosphorylated at particular sites by glycogen synthase kinase-3β (GSK-3β), which causes β-catenin to become ubiquitinated and degrade [51]. Under the presence of an aberrant COX-2/PGE2 ratio, excessive β-catenin accumulation together with elevated anti-nucleosome autoantibodies could lead to neuronal death in response to glutamate excitotoxicity (Figure 3).

## 5. Conclusions

The COX2/PGE2 ratio, when combined with anti-nucleosome autoantibodies, has a higher predictive power than direct assessment of PGE2 levels and COX-2 enzyme activity [22]. ASD patients with an abnormal COX-2/PGE2 signaling pathway have a significantly lower ratio, despite higher levels of independent variables [28]. The increased AUC of the combined ROC of COX/PGE2 and anti-nucleosome autoantibodies, along with their proven roles in impaired lipid metabolism, autoimmunity, and glutamate excitotoxicity as the disorder’s three etiological mechanisms (illustrated in Figure 3), raises the possibility that these variables could be used for an early diagnosis of ASD and certain co-morbidities seen in ASD patients.

### Limitations

A disadvantage of the present study is the imperfect sample size and variations in the number of evaluated samples caused by insufficient blood samples.

## Figures and Tables

**Table 1:** Demographic data of participants with ASD and controls.

Variables	Autistic (*N* = 40)	Controls (*N* = 42)
Age	2–12 years (7.98 ± 2.59)	2–14 years (7.83 ± 2.64)
Sex	All males	All males
Weight	29.24 ± 11.45 kg	28.81 ± 11.04 kg
Height	129.19 ± 15.73 cm	128.96 ± 16.28 cm
Family history of autoimmune	16/40 (40%)	3/42 (7%)

**Table 2:** Comparison between control group and patient group.

Parameters	Groups	*N* *	Min.	Max.	Mean ± S.D.	Median	Percent Change	*p* Value
COX/PGE2	Control	40	0.180	3.070	0.87 ± 0.67	0.601	100.00	0.001
	Patient	42	0.000	1.130	0.35 ± 0.28	0.231	39.99	
Anti-nucleosome	Control	14	0.050	5.940	1.20 ± 1.80	0.220	100.00	0.001
	Patient	32	0.050	34.130	4.43 ± 5.85	3.261	368.69	

* Variations in the amount of blood samples examined are caused by insufficient blood withdrawal samples.

**Table 3:** Correlations between COX/PGE2 with anti-nucleosome using Spearman Correlation.

Parameters	R (Correlation Coefficient)	*p* Value	
COX/PGE2 with anti-nucleosome	−0.178	0.247	N ^a^

^a^ Negative correlation.

**Table 4:** ROC results for results for patient group according to control group as a reference group.

Parameters	AUC	Cut-Off Value	Sensitivity %	Specificity %	*p* Value	95% CI
COX/PGE2	0.808	0.252	57.1%	97.5%	0.001	0.716–0.900
Anti-nucleosome	0.830	0.340	96.9%	64.3%	0.001	0.692–0.969

**Table 5:** Combined ROC results of COX/PGE2 with anti-nucleosome for patient group according to control group as reference group.

Parameters	AUC	Sensitivity %	Specificity %	*p* Value	95% CI
COX/PGE2 with anti-nucleosome	0.898	93.3%	78.6%	0.001	0.785–1.000

**Table 6:** Logistic regression (patient group).

Parameters	Regression Coefficient	Standard Error	Odds Ratio	95% CI for Odds Ratio		*p* Value
				Lower	Upper	
COX/PGE2	−6.099	2.306	0.002	0.000	0.206	0.008
Anti-nucleosome	0.600	0.268	1.821	1.078	3.077	0.025

## Data Availability

The original contributions presented in this work are included in the article; further inquiries can be directed to the corresponding author.
